# Cooperative Regulation of Non-Small Cell Lung Carcinoma by Nicotinic and Beta-Adrenergic Receptors: A Novel Target for Intervention

**DOI:** 10.1371/journal.pone.0029915

**Published:** 2012-01-12

**Authors:** Hussein A. N. Al-Wadei, Mohammed H. Al-Wadei, Hildegard M. Schuller

**Affiliations:** 1 Experimental Oncology Laboratory, Department of Biomedical and Diagnostic Sciences, College of Veterinary Medicine, University of Tennessee, Knoxville, Tennessee, United States of America; 2 Department of Preventive Medicine, Sana'a University, Sana'a, Yemen; Institut National de la Santé et de la Recherche Médicale - Institut Cochin, France

## Abstract

Lung cancer is the leading cause of cancer death; 80–85% of lung cancer cases are non-small cell lung cancer (NSCLC). Smoking is a documented risk factor for the development of this cancer. Although nicotine does not have the ability to initiate carcinogenic events, recent studies have implicated nicotine in growth stimulation of NSCLC. Using three NSCLC cell lines (NCI-H322, NCI-H441 and NCI-H1299), we identified the cooperation of nicotinic acetylcholine receptors (nAChRs) and β-adrenergic receptors (β-ARs) as principal regulators of these effects. Proliferation was measured by thymidine incorporation and MTT assays, and Western blots were used to monitor the upregulation of the nAChRs and activation of signaling molecules. Noradrenaline and GABA were measured by immunoassays. Nicotine-treated NSCLC cells showed significant induction of the α7nAChR and α4nAChR, along with significant inductions of p-CREB and p-ERK1/2 accompanied by increases in the stress neurotransmitter noradrenaline, which in turn led to the observed increase in DNA synthesis and cell proliferation. Effects on cell proliferation and signaling proteins were reversed by the α7nAChR antagonist α-BTX or the β-blocker propranolol. Nicotine treatment also down-regulated expression of the GABA synthesizing enzyme GAD 65 and the level of endogenous GABA, while treatment of NSCLC cells with GABA inhibited cell proliferation. Interestingly, GABA acts by reducing β-adrenergic activated cAMP signaling. Our findings suggest that nicotine-induced activation of this autocrine noradrenaline-initiated signaling cascade and concomitant deficiency in inhibitory GABA, similar to modulation of these neurotransmitters in the nicotine-addicted brain, may contribute to the development of NSCLC in smokers. Our data suggest that exposure to nicotine either by tobacco smoke or nicotine supplements facilitates growth and progression of NSCLC and that pharmacological intervention by β blocker may lower the risk for NSCLC development among smokers and could be used to enhance the clinical outcome of standard cancer therapy.

## Introduction

Lung cancer is the leading cause of cancer-related mortality in both men and women in the United States, with a similar trend documented globally. Industrialized regions such as North America and Europe have the highest rates [1]. The family of non-small cell lung cancers (NSCLC) consists of adenocarcinoma, squamous cell carcinoma and large cell carcinoma. NSCLC is highly resistant to existing cancer therapeutics, and the survival rate beyond 2 years is still discouraging despite recent advances in the development of novel agents that target epidermal growth factor or angiogenesis factors [2]. NSCLC accounts for about 80% of all lung cancer cases, with adenocarcinoma predominating. Smoking is a documented risk factor for NSCLC and is primarily responsible for the development of this cancer type in populations exposed to smoking [3], [4]. The nitrosated nicotine-derived carcinogen 4-(methylnitrosamino)-1-(3-pyridyl)-1-butanone (NNK)causes NSCLC in laboratory rodents [5], [6] and has been identified as a high affinity agonist for nicotinic acetylcholine receptors (nAChRs) [7], [8]. Cellular signaling in response to binding of NNK to nAChRs has been implicated in the growth regulation of NSCLC [9]. However, because the tobacco constituent nicotine is less carcinogenic compared to its potent carcinogenic derivative NNK, nicotine has attracted less interest for its role in smoking's effects on cancer growth and stimulation. It has been earlier shown that nicotine stimulates angiogenesis via binding to α7 nAChR [10], a process that may stimulate tumor growth. However, these studies have addressed direct cellular responses to single doses of nicotine or NNK, which may be termed acute exposures. Such a protocol does not mimic real-life exposure where exposure to both agents in smokers is chronic, suggesting that similar to the effects of chronic nicotine in the brain, indirect mechanisms via the nAChR-mediated production of neurotransmitters may be involved. Moreover, nicotine replacement therapy may have adverse effects on the clinical outcome of NSCLC therapy via such mechanisms.

Nicotinic receptors are constituted of alpha subunits (homomeric nAChRs) or a combination of alpha and beta subunits (heteromeric nAChRs). Initially, this receptor family was thought to be restricted to the nervous system, and the receptors' biology as well as responses to acute and chronic nicotine have been extensively studied in the brain [11]. However, more recent studies have identified nAChRs in numerous non-neuronal mammalian cells, where they regulate diverse cellular functions [12]. Research examining the role of neuro-excitatory stimuli in the brain has identified the homomeric α7nAChR as the regulator of excitatory neurotransmitters, such as glutamate, and the stress neurotransmitter noradrenaline, from which adrenaline is formed enzymatically [13], [14]; on the other hand, the heteromeric α4β2nAChR regulates the inhibitory neurotransmitter γ-aminobutyric acid (GABA) [15]. Chronic exposure to nicotine upregulates the protein expression of both receptors in the nervous system via posttranscriptional mechanisms without an increase in gene transcription [6], [16], [17]. The upregulation of α4β2nAChR protein is the response to nicotine-induced, long-term desensitization that reduces GABA production in the brain, an effect thought to cause nicotine addiction and craving [11]. By contrast, protein upregulation of the α7nAChR in the brain is not accompanied by long-term receptor desensitization, resulting in enhanced production of excitatory neurotransmitters [18].

It has been demonstrated that binding of nicotine to the α7nAChR stimulated colon cancer cells indirectly by increasing the production of noradrenaline, which in turn activated β-adrenergic receptor (β-AR) signaling [19]. Noradrenaline is the physiological agonist for the Gα_s_-coupled β-ARs, and many of its biological effects are caused by the activation of adenylyl cyclase downstream of Gα_s_ that leads to the formation of intracellular cAMP [20], [21]. Noradrenaline has strong stimulating effects, via beta-adrenergic receptor signaling, on a number of cancer types, including cancer of the colon [19], [22], [23], prostate [24], ovary [25], and pancreas [26]. It has also been shown that the selective β-adrenergic agonist isoproterenol stimulates DNA synthesis of human NSCLC cell lines [27]. A cooperative regulation seems to be at work, as NNK is not only an agonist for nAChRs [7], [8] but also for β-ARs [28] and has been shown to stimulate proliferation and migration of human NSCLC cell lines in vitro via signaling effectors downstream of β-ARs. The beta-adrenergic signaling cascade activated by NNK in these cells included the adenylyl cyclase/cAMP/CREB pathway as well as PKA-dependent transactivation of EGFR and its downstream effector, the ERK1/2 cascade [28], [29]. Such activation of cancer stimulatory β-adrenergic signaling by NNK might also be the principal regulatory mechanism involved in the action of its parent compound, nicotine, because of its documented ability to cause the release of noradrenaline and adrenaline.

In the current study, three human NSCLC cell lines (NCI-H322, NCI-H441, NCI-H1299) were used to test the hypothesis that acute or chronic nicotine-induced modulation of α7nAChR and α4β2nAChR may contribute to the development and progression of AC in smokers in a manner similar to changes in these receptors in the nicotine-addicted brain, and that these effects can be neutralized by γ-amino butyric acid or a general beta-blocker such as propranolol.

## Materials and Methods

### Cell lines and tissue culture

The human NSCLC cell line NCI-H322 (histological subtype: adenocarcinoma with activating point mutation in K-ras) was purchased from the European Collection of Cell Cultures (Health Protection Agency, Porton Down, Salisbury, UK). The NSCLC cell lines NCI-H441 (histological subtype: adenocarcinoma without activating point mutation in K-ras) and NCI-H1299 cells (non- small cell lung carcinoma which lacks p53 protein expression, histological subtype not identified) were purchased from American Type Culture Collection (Manassas, VA, USA); Cell line NCI-H1299 was purchased by us 1 month earlier and therefore did not require authentication by us. Cell lines NCI-H322 and NCI-H441were purchased by us several years earlier and were therefore authenticated by RADIL (Columbia, MO, USA) by species-specific PCR. Cells were maintained in RPMI-1640 culture medium (Gibco, Frederick, MD, USA) supplemented with fetal bovine serum (10% v/v), free of antibiotics at 37°C in an atmosphere of 5% CO_2_ [29]. Similar to the majority of human NSCLCs [30], NCI-H322 carries k-ras point mutation while NCI-H441 does not have such a mutation.

### ELISA immunoassays for the detection of noradrenaline and GABA

Neurotransmitter production mediated by nAChRs in response to a single dose of nicotine was determined by acute exposure of cells for 30 minutes to 1 µM nicotine (nicotine [-]-tartrate, Sigma, St. Louis, MO, USA) in the presence or absence of the selective α7nAChR antagonist α-bungarotoxin (α-BTX, 200 nM, Calbiochem, Gibbstown, NJ, USA) or the selective α4β2nAChR antagonist N-n-decylnicotinium iodide (NDNI 200 nM, Sigma). Chronic exposure of cells with nicotine (1 µM) was conducted for 7 days in basal medium containing heat-inactivated horse serum (Sigma) as the only additive with replacement of nicotine every 24 hours. Control cells were maintained under identical conditions for 7 days without nicotine. In order to measure modulations in the sensitivity of α7nAChR and α4β2nAChR to chronic nicotine, control cells and nicotine pretreated cells were then washed with PBS and exposed for 30 minutes in basal medium to nicotine at concentrations from 10 nM through 10 µM, and noradrenaline and GABA were analyzed by immunoassays. For determination of EC_50_ values, the data were analyzed by nonlinear regression and fitted to sigmoidal dose-response curves with variable slopes, using Prism GraphPad software (San Diego, CA, USA). For the nonlinear regression analysis of the ascending curves for noradrenaline, as a restraint for the calculation the EC_50_, the values for the untreated control cells were entered as bottom of the curve (minimum). For the nonlinear regression analysis of the descending GABA curves, as a restraint for the calculation of the EC_50_, the values for the untreated control cells were entered as top of the curve (maximum). The EC_50_ values were tested for significant differences of control versus nicotine pretreated cells, using unpaired, two-tailed *t* tests.

Measurements of noradrenaline and GABA in cultured cells were done by enzyme immunoassays according to the manufacturer's instructions (noradrenaline: 2-CAT ELISA, GABA: GABA Elisa, Rocky Mountain Diagnostic, Inc., Colorado Springs, CO, USA). Absorbance was read with an ELISA reader at 450 nm. Quantification of the samples was achieved by comparing their absorbance with a reference curve prepared with known standard concentrations of noradrenaline and GABA. Data are expressed as mean values and standard errors from triplicate samples per treatment group. Statistical significance of data was assessed by one-way analysis of variance (ANOVA), the Tukey-Kramer multiple comparison test, and two-tailed, unpaired *t* test.

### Assessment of cell proliferation by MTT assay

The colorimetric 3-(4, 5-dimethyle thiazol-2-yl)-2, 5-diphenyl tetrazolium bromide (MTT) assay (Sigma) was performed as previously described [31] to assess the concentration dependence of inhibitory effects of GABA on cell proliferation. NSCLC cells were treated with GABA (0.5 µM to 128 µM) for 72 hours. The data were then analyzed by nonlinear regression using GraphPad Prism software.

Also, an MTT assay was used to determine the effect of acute or chronic nicotine on NSCLC cells in the presence or absence of the general β-adrenergic antagonist (synonym: beta-blocker) propranolol (Sigma). The dose of nicotine used in this study was within the range of daily nicotine intake in heavy smokers [32].

### Determination of DNA synthesis by [^3^H]-thymidine incorporation assay

Analysis of DNA synthesis by [^3^H]-thymidine incorporation assay was conducted as previously described [28]. Briefly, cells were seeded into 96-well plates (5×10^3^ cells/well, triplicate wells/treatment group) in complete RPMI-1640 and allowed to settle; following 24-hour incubation in 37°C in an atmosphere of 5% CO_2_ and 99% RH, complete medium was then replaced by fresh, low-serum medium (0.1% FBS) for 24-hour starvation, at the end of which medium was replaced by fresh, low-serum medium containing [^3^H]-thymidine (0.5 µCi/well) and treatment agents (inhibitor: 1 µM propranolol, or stimulator: 1 µM epinephrine, Sigma, or a combination of 1 µM propranolol for 10 minutes' preincubation followed by 1 µM epinephrine) or vehicle in a final volume of 200 µl/well. Following an additional incubation period of 24 hours in the presence of the treatment agents in 5% CO_2_ and 99% RH at 37°C, cells were washed three times with PBS and lysed, adding 30 µl/well of 0.1 N NaOH. The plate was incubated at room temperature on a horizontal orbital microplate shaker. Free [^3^H]-thymidine was separated from incorporated [^3^H]-thymidine by vacuum filtration using a microplate harvester (Micromate 196, Packard, Meriden, CT, USA) onto backed glass fiber filters. The filters were flushed with isopropanol (200 µl/well) to fix adsorbed DNA and transferred to scintillation vials containing 3 ml counting cocktail (Bio-Safe-II, IL, USA). Radioactivity bound to the filters was measured by liquid scintillation spectrophotometry (Packard BioScience, Waltham, Massachusetts, USA). GraphPad software was used to analyze data. Means and standard errors were generated for count per minute. Significant differences between groups were assessed by ANOVA and unpaired Student *t* test.

### Protein expression analysis of nAChRs and their effectors by Western blotting

To assess the effects of one-time nicotine exposure versus chronic exposure on the expression of nAChRs subunits (α7, α4), glutamate decarboxylase (GAD65), and the mitogen-activated protein kinases ERK1/2 or the cAMP response element binding protein CREB, NSCLC cells were seeded into tissue culture dishes (100 cm^2^) containing their growth media. When the cells had reached 60–65% confluence, they were rinsed once with 1× PBS and starved of serum for 24 hours. Following removal of the media and replacement with fresh basal media, nicotine (1 µM) and cells were incubated for 30 minutes in serum-free media with heat-inactivated horse serum for 7 days. In another set of experiments, cells were seeded into 100 cm^2^ culture dishes containing their growth media until they had reached 60–65% confluence, and then cells were switched into basal media for 24-hour starvation. Following starvation, fresh basal media was added containing the treatments (1 µM nicotine, 30 minutes; 1 µM propranolol, 40 minutes; or a combination of propranolol for 10 minutes followed by co-treatment with nicotine for 30 minutes). Following treatments, cells were then washed once with cold 1× PBS followed by cell lysate collection in which the protein concentration was estimated by BCA Protein Assay (Pierce, Rockford, IL, USA). Western blotting procedure was conducted as previously described [26]. Following blocking, membranes were then incubated with primary antibodies overnight at 4°C. These antibodies included total CREB (Upstate Biotechnology, Lake Placid, NY, USA), p-CREB, p-ERK1/2 and ERK1/2 (Cell Signaling, Danvers, MA, USA), anti-α4nAChR, α7nAChR, GAD65 (Millipore, Billerica, MA, USA) and anti-beta-actin (Sigma). After incubation with horseradish peroxidase-labeled secondary antibody (anti-mouse or anti-rabbit, Cell Signaling) for 1 hour at room temperature, immunoreactive bands were detected by chemiluminescent reaction (ECL, Amersham Biosciences, Piscataway, NJ, USA) via autoradiography on Kodak Bio-Max XAR film. Relative densities of the bands were determined by image analysis using NIH SCION image analysis software. Mean values and standard errors from five densitometric readings per band were analyzed by one-way ANOVA and Tukey-Kramer multiple comparisons test or by non-parametric ANOVA and Mann-Whitney test as appropriate.

## Results

### NSCLC cells release the neurotransmitters noradrenaline and GABA in response to nicotine, and these effects are inhibited by α7 or α4 nAChR antagonists

**Figure 1:**
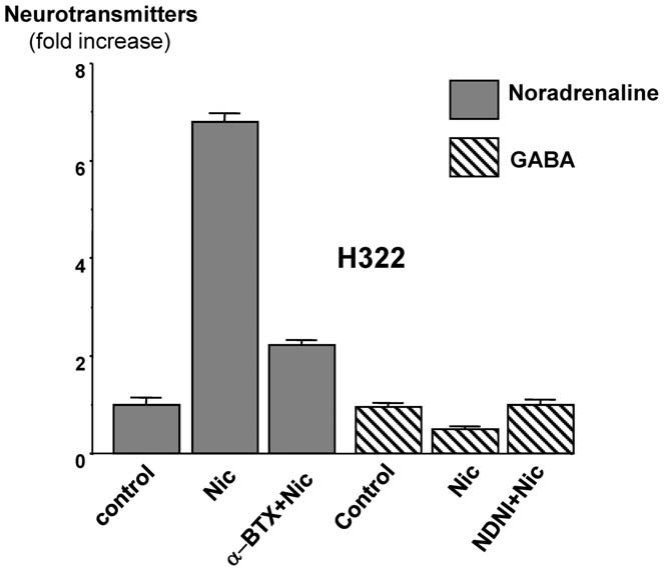
Effect of acute nicotine exposure (Nic, 1 µM for 30 minutes) on the production of noradrenaline and GABA in NCI-H322 cells. Production of noradrenaline was significantly (P<0.001) increased (ELISA immunoassay), an effect inhibited by the α7nAChR antagonist α-bungarotoxin (α-BTX, 200 nM for 30 minutes). Production of GABA was significantly (P<0.001) reduced by nicotine, a response blocked by the α4β2nAChR antagonist N-n-decylnicotinium iodide (NDNI, 200 nM). Columns are mean values and standard errors of triplicate samples per treatment group.

Exposure of NCI-H322 cells to a single dose (1 µM) of nicotine for 30 minutes caused a significant (P<0.001) increase in noradrenaline production (Fig. 1). This response was significantly (P<0.001) inhibited by the selective α7nAChR antagonist α-BTX (200 nM; Fig. 1). At the same time, nicotine significantly (P<0.01) reduced GABA production, an effect reversed (P<0.01) by the selective α4β2nAChR antagonist NMDNI (Fig. 1).

**Figure 2:**
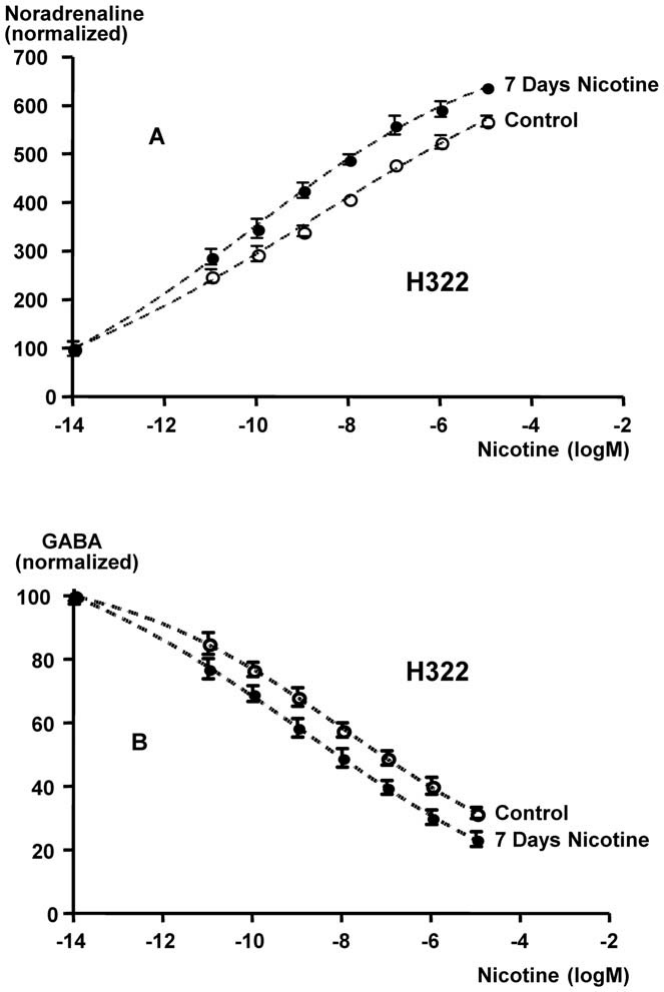
Dose-response curves of noradrenaline (A) and GABA (B) production by NCI-H322 cells acutely and chronically exposed to nicotine (1 µM). Data were generated by ELISA assays, analyzed by nonlinear regression, and fitted to sigmoidal dose-response curves. The resulting EC_50_ values were tested for statistically significant differences by an unpaired *t* test. Chronic nicotine pretreatment for 7 days significantly (P<0.001) increased the noradrenergic response while significantly (P<0.001) reducing the GABA response. Data points in the graphs are mean values and standard errors of triplicate samples.

Exposures of NCI-H322 cells for 30 minutes to ascending concentrations of nicotine established clear dose-response curves (as indicated by goodness of fit R squared values between 0.9685 and 0.97320) for the stimulating effects of nicotine on noradrenaline production (Fig. 2A) and the suppressing effect on GABA release (Fig. 2B). The noradrenergic response was significantly increased in cells pretreated for 7 days with nicotine, as evidenced by a significantly lower EC_50_ in the nicotine pretreated cells (EC_50_ for unpretreated cells: 11.2±0.10 nM; EC_50_ for nicotine pretreated cells: 1.2±0.11 nM). These findings indicate that the α7nAChR that regulates noradrenaline production was 9.2 times more sensitive to nicotine in cells pretreated for 7 days with nicotine than in unpretreated cells. In addition, the suppressing effects of nicotine on GABA production were significantly (P<0.0001) increased by chronic pre-exposure to nicotine (Fig. 2B), with EC_50_ values of 11.2±0.83 nM and 1.4±0.81 nM, respectively. These findings indicate that the α4β2nAChR was 7.9 times more desensitized by 7 days of nicotine exposure than by a single nicotine exposure for 30 minutes.

### Acute or chronic exposure to nicotine increases, and GABA or propranolol inhibits cell proliferation

**Figure 3:**
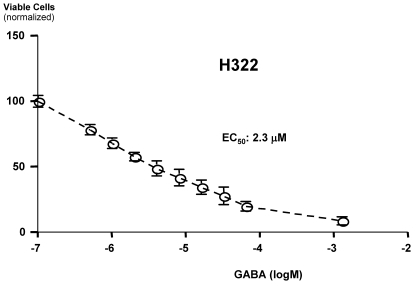
Dose-dependence of GABA treatment on tumor cell proliferation in NCI-H322 cells. Results from MTT assays were conducted in vitro. Nonlinear regression analysis fitted for a sigmoidal dose-response curve established an EC_50_ of 2.3 µM. Data points are mean values and standard deviations of five samples per GABA concentration.

**Figure 4:**
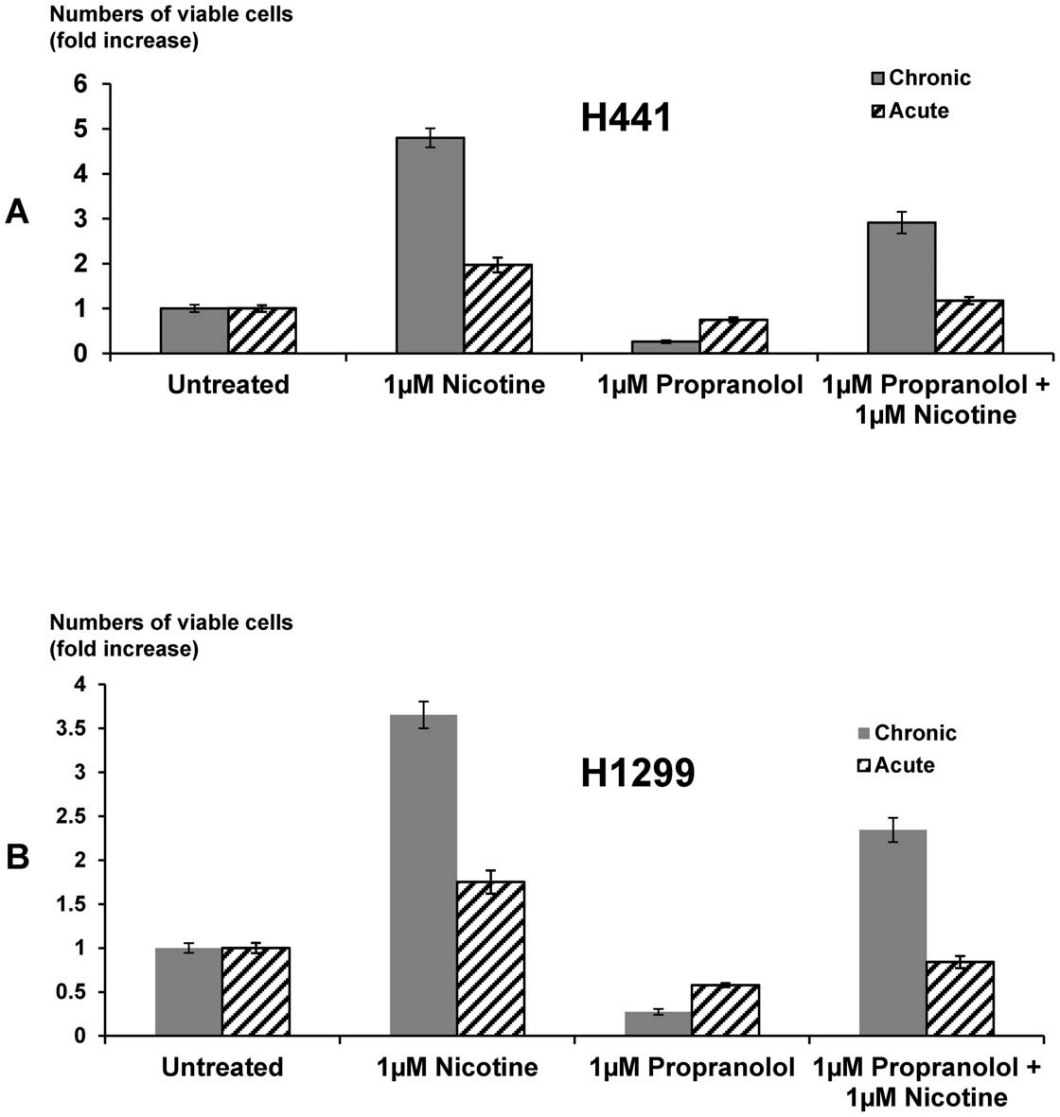
Inhibitory effects of propranolol in NCI-H441 and NCI-H1299 cells. Cells exposed to the broad spectrum antagonist of the β-adrenergic receptor propranolol (1 µM) prior to acute and chronic exposure to nicotine (1 µM) showed a significant reduction in viable cell numbers as assessed by the MTT assay. Cells were preincubated for 10 minutes with the inhibitor and then exposed for 24 hours to nicotine in the presence of inhibitor. The stimulatory response to nicotine was significantly reduced (P<0.0001). Also, the difference between the group treated with propranolol alone versus the group treated with nicotine and propranolol in both cell lines were statistically significant (P<0.001). Bars represent mean values and standard errors of three independent experiments, each with five replicate samples.

To assess the inhibitory effects of GABA on NCI-H322 cell proliferation, dose-response curves were established, using MTT assays. As exemplified in Fig. 3, the number of viable cells decreased with increasing concentrations of GABA, yielding an EC_50_ of 2.3 µM (Fig. 3). By contrast, MTT assays in NCI-H441 and NCI-H1299 cells showed that acute (30 minutes) as well as chronic (7 days) exposure to nicotine (1 µM) significantly (P<0.0001) increased the number of viable cells with chronic exposure doubling this response (Fig. 4). These acute and chronic responses of both cell lines to nicotine were significantly (P<0.0001) inhibited by the broad-spectrum β-adrenergic antagonist propranolol (1 µM; Fig. 4). The difference between the group treated with propranolol alone versus the group treated with nicotine and propranolol in both cell lines were statistically significant (P<0.001; Fig. 4).

### Exogenously-added epinephrine induces DNA synthesis in NSCLC cells

**Figure 5:**
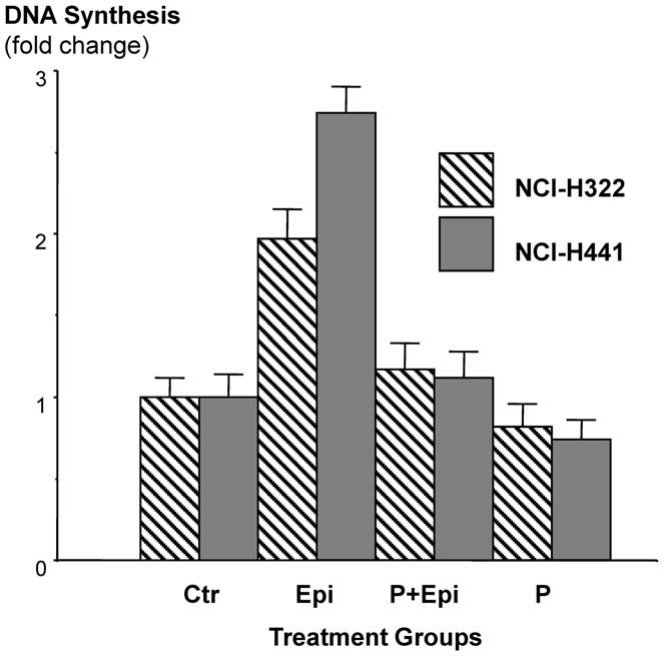
Epinephrine increased DNA synthesis, an effect blocked by propranolol. Single doses of epinephrine (1 µM) yielded a 2-fold increase (P<0.0.001) in DNA synthesis in NCI-H322 cells and a 2.8-fold increase (P<0.0001) in NCI-H441 cells ([3H]-thymidine incorporation assay). This effect was blocked and the DNA synthesis was inhibited by the broad-spectrum β-adrenergic receptor propranolol (1 µM). Data are mean values and standard errors of triplicate samples per treatment group and are expressed as normalized values relative to the control (100%) showing that variation among group medians was significantly (P<0.0001) greater than expected by chance using a non-parametric ANOVA.

Determination of [^3^H]-thymidine incorporation revealed a significant (P<0.001) increase in DNA synthesis in NCI-H441 cells (2.9-fold) when epinephrine (1 µM) was added to the culture medium and a 2-fold increase in NCI-H322 cells. This response was significantly (P<0.001) inhibited in both cell lines by the broad-spectrum β-adrenergic antagonist propranolol (1 µM; Fig. 5).

### Nicotine enhances the expression of nAChR subunits, p-CREB and p-ERK while suppressing GAD65

**Figure 6:**
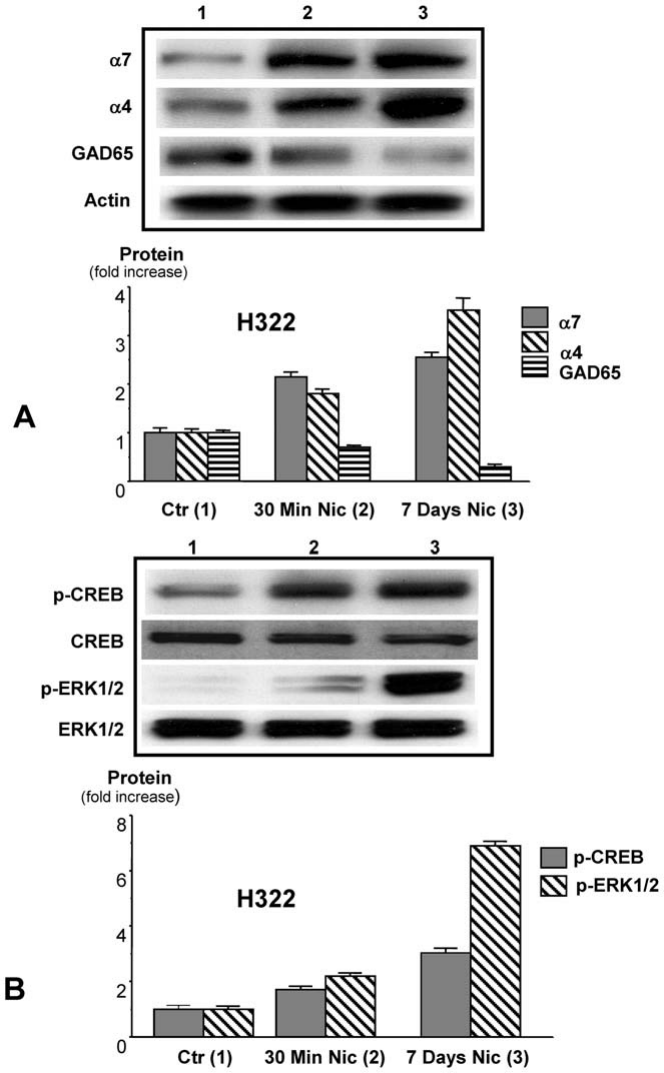
Protein expression of α7nAChR (α7), α4β2nAChR (α4), the GABA synthesizing enzyme GAD65 (A), p-CREB, CREB, p-ERK1/2, and ERK1/2 (B) in response to acute or chronic nicotine (1 µM) exposure in NCI-H322 cells. These Western blots illustrate that both nAChRs showed a significant (P<0.001) increase in protein expression after 30 minutes of nicotine exposure, an effect enhanced further by exposure to nicotine for 7 days. By contrast, the expression of GAD65 protein was significantly (P<0.001) reduced. Expression of p-ERK and p-CREB was significantly (P<0.001) increased in acutely-exposed cells. These responses were further increased (P<0.01 for p-CREB, P<0.001 for p-ERK1/2) by chronic pre-exposure to nicotine. Data in the graphs are mean values and standard errors of five densitometric readings per band calculated as ration of protein of interest over actin (A) or as ratio of phosphorylated proteins over the respective unphosphorylated proteins (B) and expressed as fold increase over untreated controls.

Protein expression of α7nAChR in NCI-H322 cells increased 2.2 fold (P<0.001) after acute nicotine exposure and 2.7 fold after chronic exposure, while expression of α4β2nAChR increased 1.8 (P<0.001) and 3.6 fold (P<0.001), respectively (Fig. 6A). Expression of the GABA-synthesizing enzyme GAD65 decreased to 0.7 and 0.3 fold, respectively, in cells treated with nicotine for 30 minutes or 7 days (Fig. 6A).

**Figure 7:**
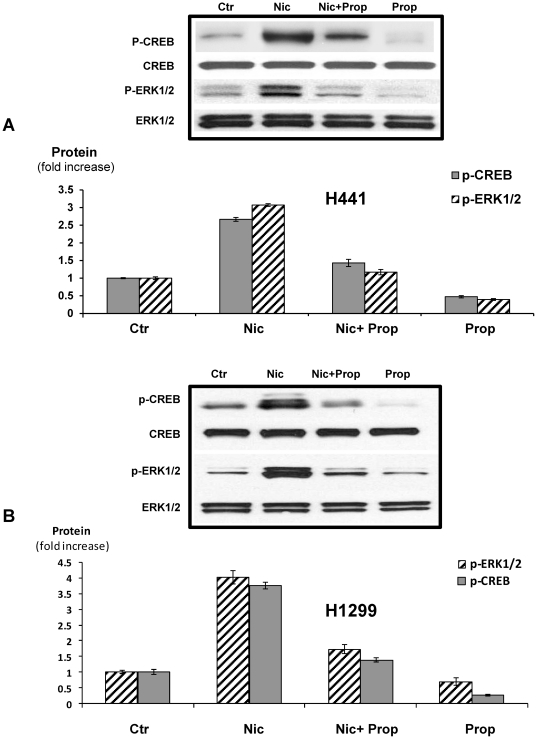
Western immunoblot analyses in NCI-H441 (A) and NCI-H1299 cells (B). Nicotine (1 µM for 30 minutes) increased the expression of p-CREB (NCI-H441:2.75 fold, P<0.001; NCI-H1299: 3.8-fold, P<0.001) and p-ERK1/2 (NCI-H441: 2.9 fold, P<0.001; NCI-H1299: 4.1 fold, P<0.001). Propranolol inhibited induction of these phosphorylated proteins (P<0.001) in both cell lines. CREB and ERK1/2 were used as housekeeping proteins to ensure equal protein loading. Columns in the graph represent mean values and standard errors of five densitometric readings per band, expressed as a ratio of p-CREB over CREB or p-ERK over ERK1/2.

Protein analysis of the unphosphorylated and phosphorylated forms of CREB and ERK revealed significant (P<0.001) increases in the phosphorylation of both proteins in cells acutely exposed to nicotine, as opposed to chronically-exposed cells. The intensity of this response was particularly noteworthy for p-ERK, which showed a 6.6-fold increase in the nicotine pretreated cells compared with only a 2.2-fold increase in the unpretreated cells (Fig. 6B). On the other hand, NCI-H441 cells treated with1 µM nicotine for 30 minutes showed a 2.75-fold increase in p-CREB (P<0.001) and a 2.9-fold increase in p-ERK1/2 (P<0.001) (Fig. 7A), while Western blot analysis of NCI-H1299 cells treated with1 µM nicotine for 30 minutes revealed a 3.8-fold increase in p-CREB (P<0.001) and a 4.1-fold increase in p-ERK1/2 (P<0.001) (Fig. 7B). Both responses were significantly reduced by 1 µM propranolol (P<0.0001) in both cell lines, as illustrated in Fig. 7.

These findings are in accord with the observed nicotine-induced increase in the stimulatory noradrenergic response and decrease in inhibitory GABA response after chronic nicotine exposure, resulting in hyperactivity of CREB and ERK.

## Discussion

Our data provide evidence, for the first time, that NSCLC cells produce their own stimulatory and inhibitory neurotransmitters and that these activities are regulated by nAChRs. The production of catecholamines by NSCLC cells observed in the current experiments is in accord with published observations that a significant number of NSCLC tumors and cell lines express the enzyme dopa-decarboxylase which mediates the conversion of L-Dopa to dopamine from which noradrenaline is formed [33]. The in vivo relevance of the current in vitro data is additionally supported by a recent report that nicotine-induced induction of NSCLC xenograft growth is reversed by the pharmacological inhibition of cAMP signaling [34]. The observed regulatory roles of the α7nAChR for the production of noradrenaline and of the α4β2nAChR for the production of GABA by NSCLC cells are analogous to the function of these receptors in the brain where noradrenaline has excitatory effects, whereas GABA acts as the main inhibitory neurotransmitter [13], [14]. Our data show that the α7nAChR-induced release of noradrenaline significantly stimulated NSCLC proliferation associated with the induction of p-ERK and p-CREB while the observed reversal of these effects by propranolol identify these and potentially other signaling proteins as the downstream effectors of β-ARs. The resulting cooperation of α7nAChR and β-ARs represents an attractive novel target for the development of more effective intervention strategies for NSCLC.

Nicotine-induced stimulation of DNA synthesis and inhibition of apoptosis were first reported in neuroendocrine lung cancer cells [35], [36]. Since then, numerous publications have described intracellular signaling in response to nAChR activation by nicotine or NNK and interpreted these effects as direct responses downstream of nAChRs [reviewed in 6]. However, a major function of nAChR-induced cation influx in the central nervous system is the regulation of excitatory and inhibitory neurotransmitters [6], suggesting that non-neuronal cells may respond in a similar fashion. This hypothesis is supported by our current data and is in accord with recent findings that hamsters with NNK-induced ACs have increased serum levels of noradrenaline and adrenaline accompanied by elevated cAMP in blood cells and tumor tissue [37]. Strong cancer-stimulating effects of noradrenaline and adrenaline, or stress that induces the release of these neurotransmitters from the adrenal medulla and sympathetic nerves, have also been demonstrated in cancer of the colon [22], prostate [24], mammary gland [38], and ovary [25], [39].

Our data show that chronic exposure of NSCLC cells to nicotine modulated the expression and function of α7nAChR and α4β2nAChR in a manner that enhanced the levels of cancer-stimulating noradrenaline while suppressing cancer-inhibiting GABA. These findings are in accord with the recently reported tumor suppressor function of GABA in NSCLC [40].

The observed upregulation of α7nAChR protein in conjunction with increased stress neurotransmitters, cAMP, and induced p-CREB and p-ERK1/2 in the current study indicate that chronic nicotine exposure rendered this receptor hyperactive. By contrast, upregulation in α4nAChR protein accompanied by suppression of GAD65 and GABA suggests that nicotine causes long-term desensitization of this receptor, resulting in a reactive upregulation of receptor protein. In support of this interpretation, our in vitro dose-response curves with nicotine revealed an upregulated noradrenergic response and down-regulated GABA response in AC cells pre-exposed for 7 days to nicotine. The observed increase in noradrenergic response was significantly greater than the nicotine-induced increase in α7nAChR protein, indicating that α7nAChR was significantly sensitized to the agonist. Moreover, the significant decrease in GABA response observed in cells chronically exposed to nicotine supports the interpretation of a desensitized α4β2nAChR that regulates GABA production in these cells. Our findings are in accord with the “paradoxical” (without long-term desensitization) upregulation of α7nACHR as opposed to the desensitization-induced upregulation of α4β2nAChR in response to chronic nicotine reported in the brain [11], [13] and demonstrate strong analogy to modulations of these receptors in the brain associated with nicotine addiction. In turn, the resulting hyperactivity of NSCLC-stimulating beta-adrenergic signaling suggests an important role of this cascade in the development and progression of this cancer in smokers. A recent electrophysiological study with immortalized human bronchial epithelial cells has shown that in vitro exposure to 1 µM nicotine for 48 hours significantly increased nicotine-induced currents [41], while investigations with immortalized human small airway epithelial cells have shown that exposure for 7 days to NNK caused a significant increase in the noradrenergic response of these cells to a nicotinic agonist [42]. Studies by PCR analyses have reported down-regulated gene expression of the α4nACHR subunit in the majority of investigated tissue samples from human ACs [43]. Although these studies were not accompanied by analyses of receptor protein expression that would detect posttranscriptional protein upregulation of the receptor in response to chronic nicotine [16], [17], they provide additional support for a reduced function of α4β2nAChR in human AC.

NNK is an agonist for nAChRs [7] as well as β-ARs [28], and in vitro studies with NSCLC cells and small airway epithelial cells have shown that beta-adrenergic receptor activation by a synthetic agonist or NNK activated the adenylyl cyclase/cAMP/PKA/CREB cascade while at the same time trans-activating EGFR and its downstream effectors in a PKA-dependent manner [27]–[29], [44]. Our current findings suggest that some of these signaling responses to NNK were triggered by the α7nAChR-activated release of noradrenaline. In addition, our findings suggest that nicotine-induced promotion of NSCLC growth reported in xenograft models [45] was at least in part caused by the nAChR-mediated release of noradrenaline and adrenaline into the systemic circulation. At the same time, production of endogenous GABA, which serves as the physiological inhibitor of this signaling cascade, was suppressed due to nicotine-induced, long-term de-sensitization of its regulatory α4β2nAChR. The simultaneous downregulation of GAD 65, as shown in our study, is in accord with observations that stress characterized by acetylcholine-induced activation of nAChR-mediated release of noradrenaline, adrenaline, and cortisol reduces GAD and GABA, resulting in an impaired GABA system [46]. While the mechanisms underlying these effects are poorly understood, the nicotine-induced increase in these stress neurotransmitters, accompanied by downregulation of GAD 65 and GABA levels in our study and their reversal by propranolol treatment is extremely encouraging, with potential for marker-guided prevention and adjuvant therapy of AC. This conclusion is supported by observations that propranolol prevented the development of NNK-induced NSCLC in hamsters that overexpressed the α7nAChR, p-CREB and p-ERK [47] while showing suppressed GAD expression [37], whereas epinephrine had strong tumor promoting effects [47]. The reversal of nicotine-induced effects by propranolol treatment in the current study is also in accord with reports that the posttranscriptional upregulation of nAChRs by nicotine is PKA dependent [48], as PKA is a substrate of cAMP formed downstream of β-ARs.

In summary, the cooperative regulation of NSCLC cells by nAChRs and β-ARs and the strong inhibiting effects of propranolol at several levels of these pathways identify agents that interfere with this regulatory cascade as promising new tools for marker-guided prevention and adjuvant therapy of a subset of NSCLCs.
